# The New City Hospital and the Board of Health

**Published:** 1857-08

**Authors:** 


### EDITORIAL.

*The New City Hospital and the Board of Health.*

Many of our readers may have learned from the daily papers of this city that a new hospital has been built, under the direction of the Board of Health, at an expense to the City Treasury of $30,000 or $40,000. A few weeks since, when the building was nearly completed, the Board of Health undertook to make the appointments necessary for putting the institution into active operation. For the purpose of rendering the hospital in the highest degree useful, and keeping the medical appointments, at least, free from political or partizan influences, the Board had been informed that on the adoption of a liberal and enlighted plan of organization, the services of a sufficient number of competent physicians and surgeons could be secured without expense to the city. When they came to make the appointments, however, they selected two distinct medical boards, each composed of eight members, viz.: six visiting physicians and surgeons, and two consulting.

One of these boards was styled the "Homœopathic Medical Board," and the other the "*Allopathic* Medical Board."

To the first was assigned one quarter of the hospital building only; but, in all other respects, they were placed on a perfect equality with each other. The editor of this Journal was *honored* with an appointment as one of the "consulting physicians to the *Allopathic* Medical Board."

This honor, however, was promptly declined, and the Board of Health informed that I belonged to no special *pathy* in medicine, and that if they wished to make use of the sick poor of this city to test the merits of the various *pathys* and *isms* of the day, they must do it without my assistance. This caused much fluttering on the part of the homœopathists and their friends, and called down upon myself no little personal censure. Meantime the Board of Health seem to have come to a stopping place. What they will ultimately do time alone will determine; but I shall have more to say on the subject hereafter.

